# Urdu version of Oswestry disability index; a reliability and validity study

**DOI:** 10.1186/s12891-021-04173-0

**Published:** 2021-03-29

**Authors:** Fareeha Amjad, Mohammad A. Mohseni-Bandpei, Syed Amir Gilani, Ashfaq Ahmad, Muhammad Waqas, Asif Hanif

**Affiliations:** 1 Faculty of Allied Health Sciences, Department University Institute of Physical Therapy, The University of Lahore, Lahore, Pakistan; 2 Pediatric Neurorehabilitation Research Center, University of Social Welfare and Rehabilitation Sciences, Tehran, Iran; 3 Faculty of Allied Health Sciences, International Linkages, University of Lahore, Lahore, Pakistan

**Keywords:** Disability, Validity, Translations, Reliability

## Abstract

**Background:**

Oswestry Disability Index (ODI) is broadly used in clinical and research settings for assessing the disability level in patients with lumbar radiculopathy but it has not been translated into Urdu language according to the pre-established translation guidelines as well as the validity and reliability of ODI Urdu version has not been tested yet. The aim of this study was to translate ODI in native Urdu language (ODI-U) according to recommended guidelines and to measure its psychometric properties in Urdu speaking patients suffering from lumber radiculopathy.

**Methods:**

Out of 108 participants, 54 were healthy (who filled ODI-U) and 54 were patients of lumber radiculopathy. The patients were administered through ODI-U, visual analogue scales for disability (VAS disability), pain intensity (VAS pain) and SF-36 at baseline and after 3 days. Reliability was investigated through test-retest method, internal consistency, standard error of measurement (SEM) and smallest detectable change (SDC). ODI-U was assessed for exploratory factor analysis, construct (convergent and discriminative) validity and content validity. Alpha level < 0.05 was considered statistically significant and psychometric standards were evaluated contrary to priori hypothesis.

**Results:**

ODI-U revealed excellent test-retest reliability for total score (ICC_2,1_ = 0.95) and for all item (ICC_2,1_ = 0.72–0.98). Cronbach’s alpha of 0.89 showed excellent internal consistency and moderate correlation between ODI-U total score and each item through spearman’s correlation coefficient (*r* = 0.51–0.76). One factor structure was created, explaining 52.5% variance. There was no floor and ceiling effect of total ODI-U score. Content validity was assessed through conducting interviews with patients and incorporating expert’s opinions. The discriminative validity was measured by independent sample t-test, where significant difference between healthy and patients (*P* < 0.001) was observed. The convergent validity was evaluated through Pearson’s correlation showing moderate positive correlation of ODI-U with VAS pain (*r* = 0.49) and VAS disability (*r* = 0.51) but moderate negative correlation with all SF-36 domains (*r* = − 0.43to − 0.63).

**Conclusion:**

ODI-U showed adequate psychometric properties. ODI-U was found to be a reliable and a valid tool to measure the level of disability in Urdu-speaking patients with lumber radiculopathy.

**Supplementary Information:**

The online version contains supplementary material available at 10.1186/s12891-021-04173-0.

## Background

Musculoskeletal disorders are leading medical problem over the globe and are one of the most frequent reasons of disability [1, 2]. Among these musculoskeletal disorders low back pain (LBP) is the 5th leading cause of patient’s visit to the clinics or hospitals [1, 3]. In western countries, the disability associated with LBP is of great concern these days as in the US, about 6.5 million of general population is bed ridden due to LBP [1, 2]. Similarly, in Pakistan the burden of LBP is also high. The prevalence of LBP in workers of different organizations of Pakistan ranges from 52.4–74.3% [4–7]. Moreover, in a tertiary care hospital of Pakistan it was found to be 78.4% [8]. Sometimes LBP may lead to Lumbar radiculopathy [9]. Due to irritation of lumbar nerve roots, pain radiates in lower limbs which is defined as lumbar radiculopathy [10].

There are numerous outcome measures for assessing LBP including Roland Morris Disability scale (RMDQ) [11], Oswestry disability index (ODI) [12], Quebec back pain disability scale [13], Waddell disability index [14] SF-36 [15] and VAS disability [2, 16]. Among all these scales ODI is considered here to be studied which is one of the most frequently used, reliable and valid tool to measure disability and pain in patients with LBP and lumber radiculopathy.

ODI is considered to be a gold standard self-reported outcome measure tool to evaluate quality of life and disability level after lumber radiculopathy [17–20]. It was designed by Fairbank JC in 1980s with various adaptations over the years and the final Version 2.1 was then created [21, 22].

According to ODI website information, this questionnaire is available in 29 languages [23, 24] but the Urdu Version of original ODI is not yet available or published. Therefore, the aim of this study was to translate original ODI into the native Urdu Language, to interpret its psychometric properties and to assess the reliability and validity of translated version.

## Methodology

This cross-sectional study was conducted over a period of almost 2 years and data was collected from October 2018 to October 2019. The study was divided into 2 stages: 1) Translation and cultural adaptation 2) Psychometric testing of ODI Urdu version.

### Stage I: translation and cultural adaptation

Permission for translation of original ODI (Additional file 1) through Mapi Research Trust by signing an addendum. The guidelines of Guillemin and Beaton (1993) [25, 26] and COSMIN guidelines [27] were used for the translation and cultural adaptation of original ODI. This process involves five steps:

#### Step 1: forward translation

Two independent native linguistic translators who were experts of both English and Urdu language have independently translated ODI from English to Urdu. The first translator was qualified in English linguistic while other translator was senior physical therapist. These two translators were blinded from each other and were requested to conceptually translate ODI instead of emphasizing on word to word translation.

#### Step 2: synthesis of translation

The discrepancies between two translations i.e. translation 1 (T1) and translation 2 (T2) were discussed by a four person committee. This committee involved an independent physical therapist, main author and both of the translators. They have created a new Urdu version (T12) from T1 and T2.

#### Step 3: reverse translation

The reverse translation of T12 was performed by two independent native translators. These translators have produced as reverse translation 1 (RT1) and reverse translation 2 (RT2). Both of the translators were blinded to the original ODI version.

#### Step 4: review of the expert committee

The expert review committee of authors including all of the translators and an expert senior physical therapist highlighted, removed and edited the conflicts and errors in translated versions of ODI. After teamwork of review committee a pre-final Urdu version of ODI was produced.

#### Step 5: testing of pre-final version

**Fig. 1:**
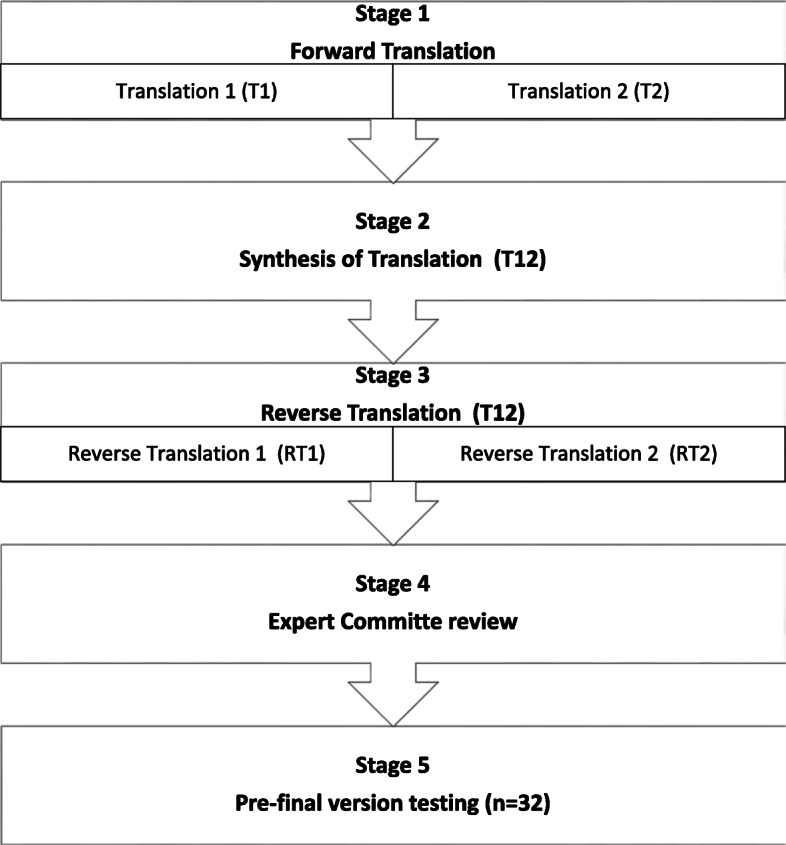
Flow Chart of Translation and Cultural Adaptation

The pre-final version of ODI was randomly distributed among 32 patients of lumbar radiculopathy and they were asked to highlights any understanding difficulties in wording and layout of the questionnaire. Patients were also encouraged to identify the ambiguous words. Final version of ODI was formulated after considering the patient’s feedback and expert committee opinion. Figure 1 is showing the flow chart of whole translation process.

### Stage II: psychometric testing

The psychometric testing of Urdu version of-ODI (ODI-U) was done according to COSMIN guidelines [27]. The total sample size was 108, out of which 54 were healthy participants and 54 were diagnosed patients of lumbar radiculopathy. The data was collected after Institutional review board (IRB) approval from The University of Lahore teaching hospital, department of physical therapy. All methods were performed in accordance with the relevant guidelines and regulations. Before data collection the informed written consent was also taken from all the participants. The inclusion criteria was married male and female of age range between 25 and 55 years, those who were able to read and speak the native Urdu language, pre-diagnosed patients of lumbar radiculopathy by physician or neuro-surgeon and fifty four healthy subjects (based on their BMI) were also age and sex matched with patients of lumbar radiculopathy. All of the healthy subjects were recruited from the staff of University of Lahore. Patients or subjects who were excluded from the study were pregnant females, having any surgery of lumber region, recent fracture or dislocation, spinal tumors, inflammatory diseases, infections in the intervertebral disc and subjects with psychological disorders.

The Reliability of final ODI-U version was measured by test re-test method across two repeated measures (1st measurement and 2nd measurement), internal consistency and agreement of repeated measurements. Meanwhile the content and construct validity were also assessed. Two types of construct validity were studied i.e. discriminative validity and convergent validity.

### Data analysis

The data analysis was carried out on IBM SPSS 21 software. *P*-value less than 0.05 (typically < 0.05) was considered to be statistically significant. The values of psychometric properties were verified through a priori hypothesis. Descriptive statistics was used to study the participant’s characteristics.

#### Reliability

The reliability of ODI-U version was tested among 54 patients of lumber radiculopathy. The sample size for reliability testing was calculated by using power calculation method which is a previously developed method to determine the sample size of reliability studies [28]. The patients were asked to complete ODI-U, VAS pain, VAS disability and and 36-item short form survey (SF-36) during their first visit. Other demographic details were also documented. After 3 days, the same patients were re-tested in the same way by completing ODI-U, VAS pain, VAS disability and SF-36. Any type of treatment was not given to the patients during this period.

The test-retest reliability was assessed by calculating intra-class correlation coefficient (ICC_2,1_) using Two-Way Mixed analysis of variance [27]. ICC values are between 0 and 1. For estimating ICC, the reliability could be poor, moderate, good and excellent with values < 0.5, between 0.5–0.75, between 0.75–0.9 and > 0.90 respectively [29–31]. The internal consistency of ODI-U was measured through cronbach’s alpha values and item total correlation. Internal consistency is considered acceptable when alpha value exceeds 0.70 [32]. or is between 0.70–0.95 [33]. The item-total correlation was calculated through Spearman’s correlation coefficient which shows the relationship strength between each item and total score of ODI-U minus the score of the item being investigated [34]. The strong relationship between two variables is considered when r value is greater than 0.7 [35]. The greater the value of the coefficient, the stronger is the correlation between the item and the total score which ensure that the scale is internally consistent [36]. Spearman rank correlation coefficient values were interpreted as little or no relationship, fair, moderate and excellent relationship with values < 0.25, 0.25–0.50, 0.50–0.75 and ≥ 0.75, respectively [37, 38]. Agreement of repeated measurements was calculated through Standard error of measurement (SEM) and smallest detectable change (SDC). The formulas used to calculate SEM and SDC are SEM = SD × √ 1 – ICC [37] and SDC = 1.96 × √2 × SEM [39], respectively. The instrument is considered more reliable if the value of SEM is less [38]. SEM values ≤2.15–6.5 [38, 40–45] and SDC values between ≤6–13.7 [38, 46–48] were considered to be acceptable. The Bland and Altman plot was used to measure the degree of within subject variation and limits of agreement with 95% confidence intervals [49]. For evaluating the agreement between ODI-U total scores at two different occasions, the limits of agreement were obtained by plotting the difference between total score of first and second measurement against the average of these two measurements, for each patient.

#### Factor analysis

Factor analysis is used to decide that either the items of an instrument form one or more than one dimensions [50, 51]. Factor analysis was executed through varimax rotation by means of principal component factor analysis. Using eigenvalues > 1, clusters of items were recognised [48]. Factor loading value ≥0.4 was assumed to be acceptable [50]. KeiserMeyer-Olkin test and Bartlett’s test of sphericity were performed for analysing that either correlation was adequately large for implementing factor analysis [52]. The cut off value for Kaiser-MeyerOlkin Measure (KMO) of Sampling Adequacy is > 0.50 [53]. The KMO values above 0.50 were considered acceptable. Previous translation studies of ODI into different languages have shown one or two factor structure of ODI [3, 41, 48]. Priori hypothesis was also not established about the ODI-U principal factor structure in the previous studies.

#### Floor and ceiling effect

The extent of floor and ceiling effect as well as the completeness of question response was calculated. It was predicted that there would be < 5% missing questions from total answers of all participants and no floor and ceiling effects will be observed [38]. As recommended by McHorney and Tarlov, the floor and ceiling effects were considered to exist if > 15% participants have attained the minimum or maximum possible total score [38, 54] [55, 3, 56]. The floor and ceiling effects were measured by calculating the number of respondents who have recorded the lowest and highest score on ODI-U, respectively [38, 46].

#### ,V.alidity

For validity of ODI-U, one hundred and eight participants were recruited. Out of which 54 were healthy subjects and 54 were patients of lumber radiculopathy. The sample size for validity study was estimated through rule of thumb i.e. a ratio of minimum 10 participants per item [57].

The **content validity** was assessed through testing the pre final version by conducting interviews with thirty two patients of lumber radiculopathy and by incorporating the expert opinion during the stage I i.e. Translation and cultural adaptation stage of the study. In order to attain satisfactory content validity, the new translated version must be adequately adapted according to the existing cultural and linguistic expression [25, 44, 58].

Two types of **construct validity** were studied i.e. discriminative validity and convergent validity. The discriminative validity was measured by calculating the difference in ODI-U total score between healthy participants and lumber radiculopathy patients by applying independent sample t-test. It was assumed that a significant difference in total score of ODI-U would be found between two groups. The convergent validity was measured through Pearson’s correlation (r) by correlating the new translation with VAS disability, VAS pain and SF-36. VAS pain and VAS disability are simple to use and easy to understand by the patients and have also been used in the previous translation studies for evaluating the convergent validity of ODI [2, 55, 59, 60]. However, SF-36 is a generic outcome measure which measures health across two dimensions (physical and mental). It has eight domains including physical function (PF), role physical (RP), Bodily pain (BP), social functioning (SF), role emotional (RE), Mental Health (MH), General Healthy (GH) and vitality (VT). SF-36 has been extensively used in previous cross-cultural reliability and validity studies of ODI [20, 41, 43, 44, 61]. Pearson correlation coefficient was interpreted as very weak correlation, weak correlation, moderate correlation, strong correlation and very strong correlation with values of 0.00 to 0.19, 0.20 to 0.39, 0.40 to 0.69, 0.70 to 0.89 and 0.90 to 1, respectively [62, 63]. It was hypothesised that there would be a moderate positive correlation of ODI-U with VAS pain and VAS disability but a moderate negative correlation of ODI-U with all SF-36 domains [20, 64, 65]. If 75% of results matched with hypothesis the validity was considered to be good [56].

## Results

### Translation and cultural adaptation

Out of thirty-two patients of lumbar radiculopathy, there were nine participants who did not answer to item 8, stating that the question was not linked to them as it was associated to sex life. To remain closer to original version of ODI, any kind of changes were avoided while translating it. The general impression of patients to ODI-U was that it was easy to understand and complete the given instructions and questionnaire items. All the items were related to underlying condition of the patient. Therefore, after performing pre-test of ODI-U, no major changes were made in it.

### Psychometric testing

In order to assess the psychometric properties of translated ODI, fifty-four male and female patients of lumber radiculopathy were enrolled in the study. Meanwhile, fifty-four healthy subjects were also recruited who were age and sex-matched to the enrolled patients. The patients were followed up after 3 days without receiving any treatment for reliability analysis but the healthy subjects were not followed up. The demographic characteristics of the participants are presented in Table 1.

**Table 1:** Participant Characteristics

***Variables***	***Patients (n = 54)***	***Healthy (n = 54)***
***Age (Years)***	41.24 ± 14.80	38.22 ± 11.45
***Gender (Male/Female)***	30/24 [55.6%/44.4%]	31/23 [57.4%/42.6%]
***VAS Pain (1st measurement)***	5.75 ***±*** 1.98	N/A
***VAS Pain (2nd measurement)***	4.96 ***±*** 2.12	
***VAS Disability (1st measurement)***	4.12 ***±*** 1.79	N/A
***VAS Disability (2nd measurement)***	3.22 ***±*** 1.74	

### Reliability

**Fig. 2:**
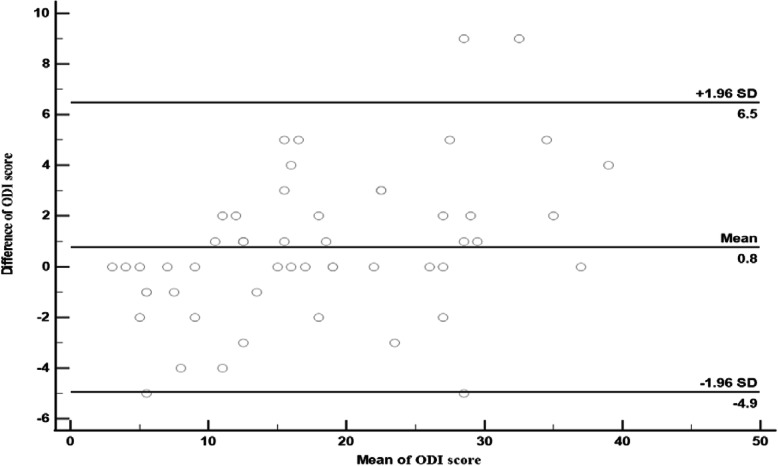
Bland-Altman Plot for Assessing the Limits of Agreement and With-In Subject Variation. ±1.96 SD indicates 95% limits of agreement

The reliability properties as well as the mean and standard deviation of all questions and total score of ODI-U are summarized in Table 2. With 54 respondents, the Urdu-ODI showed excellent test-retest reliability of each item (ICC_2,1_ = 0.72–0.98) and total ODI-U score (ICC_2,1_ = 0.95). Excellent internal consistency of ODI-U was obtained as Cronbach’s alpha value was 0.89 (**α** = 0.89) [2]. Item total correlation values ranged between 0.51–0.76 which is also confirming that ODI-U is internally consistent. SEM and SDC of all items ranged between 0.24–0.98 and 0.65–2.0 respectively. However, for ODI-U total scores SEM and SDC were 2.14 and 5.93 respectively. Figure 2 is showing the Bland-Altman plot indicating within-subject variation and limits of agreement. The systematic bias was very small, as the mean difference (d) was very close to the zero [mean difference (d) = 0.8] and the limits of agreement (LOA) ranged from − 4.9 to 6.5. The score of fourteen (25.9%) participants were totally unchanged. However, the scores of only four (7.4%) participants were out of agreement limits. The Bland and Altman plot showed strong agreement between the scores of two occasions with minimal within-in subject variation, supporting the ICCs obtained. The standard error of measurement (SEM) was 2.14, providing the “smallest detectable change” (MDC95%) of around 6 points, i.e. the smallest change in an individual’s score required to label as a “real change” (with 95% confidence) above and over the measurement error.

**Table 2:** Agreement of Repeated Measurements, Test Retest Reliability, Internal Consistency and Item Total Correlation Values for ODI-U (*n* = 54 Patients)

***ODI***	***First Measurement***	***Second Measurement***	***SEM***	***SDC***	***ICC (95% CI)***	***Cronbach’s Alpha***	***Item Total correlation***
	***Mean ± SD (n = 54)***	***Mean ± SD (n = 54)***					
***Question-1***	2.09 ± 1.08	2.04 ***±*** 1.06	0.28	0.77	0.93 (0.88–0.96)	NA	0.68
***Question-2***	1.76 ***±*** 1.50	1.78 ***±*** 1.55	0.26	0.73	0.97 (0.95–0.98)	NA	0.75
***Question-3***	1.89 ***±*** 1.49	1.91 ***±*** 1.26	0.73	2.02	0.72 (0.56–0.83)	NA	0.74
***Question-4***	1.70 ***±*** 1.57	1.70 ***±*** 1.54	0.34	0.96	0.95 (0.92–0.97)	NA	0.76
***Question-5***	2.02 ***±*** 1.73	1.76 ***±*** 1.54	0.76	2.10	0.79 (0.67–0.87)	NA	0.58
***Question-6***	2.16 ***±*** 1.68	2.02 ***±*** 1.62	0.66	1.83	0.84 (0.75–0.91)	NA	0.65
***Question-7***	1.29 ***±*** 1.17	1.24 ***±*** 1.26	0.32	0.88	0.93 (0.88–0.96)	NA	0.52
***Question-8***	1.51 ***±*** 1.55	1.37 ***±*** 1.36	0.48	1.34	0.89 (0.81–0.93)	NA	0.51
***Question-9***	1.81 ***±*** 1.44	1.72 ***±*** 1.34	0.66	1.84	0.77 (0.64–0.86)	NA	0.62
***Question-10***	2.11 ***±*** 1.69	2.05 ***±*** 1.68	0.24	0.65	0.98 (0.96–0.99)	NA	0.62
***Total Score***	18.37 ± 10.18	17.59 *±* 8.93	2.14	5.93	0.95 (0.92–0.97)	0.89	NA

### Factor analysis

The factor structure of ODI-U was evaluated through factor analysis. The Kaiser-Meyer-Olkin (KMO) measure of sampling adequacy showed that the KMO value was adequately high (0.89) and the Bartlett’s test of sphericity was found to be significant (*P* < 0.001). A one factor structure of ODI-U was established based on eigenvalues > 1. The eigenvalue of first factor was 5.25 explaining 52.5% variance. Table 3 is showing the factor loading of all items.

**Table 3:** Factor Loading Values

***ODI-U Items***	***Factor 1***
***Walking***	0.83
***Personal Care***	0.82
**Lifting**	0.81
***Pain Intensity***	0.76
***Standing***	0.72
***Traveling***	0.70
***Social Life***	0.69
***Sitting***	0.66
***Sleeping***	0.61
***Sex Life***	0.59

### Floor and ceiling effect

In Table 4 descriptive statistics, completeness of item response and floor and ceiling effect is tabulated. Descriptive statistics showed that mean score of items ranged from 1.12–1.65. There were 5 (4.6%) participants with 1 missing response to item 8 which was about sex life. Missing answers of questions presented < 5% of total 760 ODI-U items. Out of 108 participants no one has attained the highest or lowest expected total score. For ODI-U total score, the percentage of respondents scoring highest score (ceiling effect) and the percentage of respondents scoring lowest score (floor effect) is zero. However, for the individual items the ceiling effect ranged between 0.9–6.5% and the floor effect ranged from 19.4 to 39.8%. No floor and ceiling effect of ODI-U total score were observed.

**Table 4:** Descriptive Data, Distribution of Responses and Floor and Ceiling Effect (*n* = 108)

***ODI-U***	***Mean***	***S.D***	***Lowest Score***	***Highest Score***	***Missing responses to an item***	***Floor (%)***	***Ceiling (%)***
***Pain Intensity***	1.56	1.16	0	4	0	19.4	6.5
***Personal Care***	1.28	1.31	0	5	0	36.1	1.8
**Lifting**	1.37	1.31	0	5	0	30.5	2.7
***Walking***	1.25	1.34	0	5	0	35.2	4.6
***Sitting***	1.41	1.52	0	5	0	37	4.6
***Standing***	1.65	1.46	0	5	0	25	3.7
***Sleeping***	1.12	1.05	0	5	0	28.7	0.9
***Sex Life***	1.16	1.33	0	5	5	39.8	0.9
***Social Life***	1.32	1.30	0	5	0	34.3	0.9
***Traveling***	1.53	1.44	0	5	0	25	6.5
***Total Score (0–50)***	13.89	9.57	1	41	NA	0	0

### Validity

As shown in Table 5, there was a significant difference in ODI-U total scores between healthy participants and patients (*P* < 0.001) demonstrating significant construct (discriminative) validity. However, the construct (convergent) validity between ODI-U and other scales was moderate when calculated through Pearson’s correlation coefficient. A moderate positive and significant correlation was observed between ODI-U and VAS pain (Pearson’s correlation coefficient = 0.49, *P* < 0.001), a moderate positive and significant correlation was found between ODI-U and VAS disability (Pearson’s correlation coefficient = 0.51, *P* < 0.001). However, a moderate negative and significant correlation was found between ODI-U and all domains of SF-36 (Pearson’s correlation coefficient = 0.43–0.63, *P* < 0.001,all) except for vitality which has moderate negative but insignificant correlation with ODI-U (Pearson’s correlation coefficient = − 0.59, *P* = 0.12).

**Table 5:** Testing ODI Construct Validity

***ODI-U Total Score Difference***	***Mean ± SD***	***P -Value***
Patients	18.37 ± 10.18	< 0.001
Healthy	8.95 ± 5.74	
Difference (SE)	9.41 ± 1.65	
**Pearson’s Correlation Coefficient**	***r***	***P -Value***
Between ODI-U and VAS _pain_	0.49	< 0.001
Between ODI-U and VAS _disability_	0.51	< 0.001
Between ODI-U and SF-36 Domains		
ODI-U and Physical Functioning (PF)	−0.57	<.001
ODI-U and Role Physical (RP)	−0.61	<.001
ODI-U and Bodily pain (BP)	−0.54	<.001
ODI-U and General Health (GH)	−0.43	<.001
ODI-U and Vitality (VT)	−0.59	0.12
ODI-U and Social Functioning (SF)	−0.63	<.001
ODI-U and Role Emotional (RE)	−0.55	<.001
ODI-U and Mental Health (MH)	−0.48	<.001

## Discussion

To the author’s knowledge, it was the first research study that not only translated and cross culturally adapted original version of ODI in Urdu language according to recommended guidelines but also observed the validity and reliability of ODI-U. In 2014, Ibrahim et. Al [66] translated ODI into Urdu language and applied it in an interventional study in which caudal epidural injections were given to patients with lumbar prolapsed disc. Although assessment was made through ODI-U but permission of translation from developers was not taken as well as reliability and validity of translated version was not assessed through pre-established guidelines. However, in the present study first of all the permission for translation was taken from the developers of ODI and the process of translation was carried out according to the recommended guidelines.

In 2019, Muhammad Baber Ikram and Rana Bilal Naeem [67] cross culturally adapted the Modified form of Oswestry Disability Questionnaire (MODI) and assessed only the test-retest reliability (ICC = 0.91) of MODI. On the contrary, the present study had translated the original ODI instead of MODI and assessed all types of reliability including the test-retest reliability; internal consistency and agreement of repeated measurements. Exploratory factor analysis was also performed as well as convergent, discriminative and content validity was also measured. By pre-defined hypothesis the psychometric properties of ODI-U were proven. The results of this study showed that ODI-U has excellent reliability and fair to moderate validity.

Adaptation process revealed that ODI-U was effectively translated according to pre-established guidelines. By the use of careful language and taking the consensus decisions of expert review committee, all the hurdles faced during the adaptation process were efficiently handled. In the clinical settings, ODI-U was observed to be an easy and simple to use.

The current study has recruited more males 61 (56.45%) than females 47 (44%) which is comparable to the previous studies (54–80%) [38, 65, 68]. However many studies have recruited more females (52–66%) than males [1, 20, 23, 41, 48]. In present study, mean age of patients was 39.7 years, that is quite similar (36–40 years) to the previous research studies [1, 20, 38, 68] but in contrast few studies have enrolled patients with slightly higher mean age (40–52 years) [23, 41, 48, 65].

In the present study, internal consistency was found to be excellent with 0.89 Cronbach’s alpha value, which is also in the range of results of previous studies (0.75–0.99) [3, 42, 55, 59, 69–73] The item total correlation between single item and total score of ODI-U ranged between 0.51–0.76 which is quite similar to the findings of German version of ODI (0.58–0.72) [74]. However, in the study of Liu et al. (Chinese ODI version) the item total correlation was reported as slightly high (0.59–0.83) [75]. Excellent test re-test reliability (ICC = 0.95) was found in this study which is comparable to the previous translation studies with excellent test retest results [20, 42, 43, 48, 55, 61, 70, 75–77] and the original ODI English version (ICC = 0.91) [78]. However, the ICC value was found to be less in Russian (0.7), Norwegian (0.88) and Marathi (0.88) ODI versions [23, 59, 64]. Baradaran et al. [79] showed low ICC value i.e. 0.68. Test re-test values may vary due to interval variation used to find out the test retest reliability. To ensure the minimum changes in patient’s condition, the present study used three-day interval similar to the previous studies which also used less test retest interval [55, 80, 81]. Dawson et al. recommended 2–3 days interval to avoid patient’s condition changes [82]. On the contrary, 1–2 week interval was recommended by Deyo et al. [83] and Terwee et al. [56] to minimize the memory effects.

The Bland-Altman plot showed very minimal systematic bias i.e. 0.8 points, which is comparable to the previous ODI translation studies. The limits of agreement (LOA = -4.9–6.5) was found to be narrow than the Tamil (LOA = 12.41 to − 7.15) and Chinese (LOA = − 12.5 to 13.7) version of ODI [2, 44]. Based on the results of present research study, a change of approximately 6 points on ODI-U (0–50 scale) is required to be considered as a “real change”. This SDC value is slightly lower than the values observed in the previous studies i.e. 9 point [54], 10 point [84], 11 point [64], 13 point [40] and 17 point [54].

Previous studies evaluated factor analysis of ODI in different languages. Many studies have found two factor structures [3, 41, 61, 74, 81] while Monticone et al. have found one factor structure of ODI [48] explaining 45% of variance which is comparable to present study having one factor structure with slightly high variance i.e. 52.5%. The percentage of variance is comparable to Finnish [3] Spanish [61] and Arabic [81] version, where two factor structure explained 51, 55.6 and 58.1% of variance, respectively. However, the Croatian version found two factor structures explaining higher variance of 82.7% [41]. Compared to the previous studies, there are few differences in factor structure of present study which may be influenced by cultural differences.

It was observed that the ‘sex question’ is being omitted in some studies [12] as it is unacceptable for some cultures. In order to compensate question eight which is about sex life, the present study had enrolled only the married individuals. Still there were 5 (4.6%) participants who did not complete question about sex life; the remaining questions were answered by 100% of the participants. The appropriate reason for not answering the item eight was not mentioned by those participants. It was assumed that question 8 was not missed due to any problem in translation so; modification of this section was not needed. In contrast, the previous studies did not specify the married individuals. Therefore previous studies have reported more participants (12.9, 14.7, 19, 23, 29%) who did not complete item 8 [3, 41, 43, 61, 74].

The only noticeable difficulty in the translation process was about the description of walking distance in item 4. In the original version of ODI the British Imperial System was used and the distances were described as 1 mile, ½ mile and 100 yards. In general, it is difficult for a patient to understand the exact distances and answer it correctly. Therefore, the description of distance should be simple and not be divergent enough. The British Imperial System is also understandable in Pakistan therefore the description of distance was kept in “miles” but to avoid divergence and keeping the scale in homogenous pattern the “100 yard” was converted to “a quarter of a mile”.

In the present study, no floor and ceiling effect was found for the total score of ODI-U which is comparable to Croatian version of ODI [41]. On the contrary, the Chinese version of ODI have reported minor floor effect (0.6%) but no ceiling effect (0%) [44]. However for individual items some floor and ceiling effects were observed in the present study. The floor effects of personal care, walking, sex life and social life was found to be higher i.e. 36, 35, 39, 34% which is comparable to finnish version of ODI with 43, 43, 35 and 25% floor effect, respectively. Along with the above mentioned items, the floor effect of sitting (37%) was also higher in the present study.

To the best of author’s knowledge, the previous studies have not assessed the construct (discriminative) validity of ODI by comparing the healthy participants and patients. However, in the present study the significant difference between ODI total score of patients and healthy subjects was detected which is showing good construct validity of ODI-U. Moreover, similar to the previous studies ODI-U revealed positive correlation between ODI-U total score and VAS disability [2] as well as VAS pain [59, 72, 80]. Effect size of correlation (r) was moderate between ODI-U and VAS pain (*r* = 0.49) and VAS disability (*r* = 0.51). The effect size of correlation between ODI-U and VAS pain (*r* = 0.49) was found similar to some previous studies [55, 59, 61, 71, 72]. On the contrary, pearson’s correlation coefficient was lower (*r* = 0.370) in Turkish and Polish versions [42, 85] but higher (*r* = 0.54–0.78) in other ODI versions [23, 43, 48, 69, 76]. Furthermore, the pearson’s correlation between ODI-U and VAS disability (*r* = 0.51) was lower than the Tamil version (*r* = 0.81) [2]. The ODI-U has shown moderate negative correlation between ODI-U and SF-36 domains which ranged from *r* = − 0.43to-0.63. These results are closely comparable to the Croatian version (r = − 0.35to-0.62) [41] as well as to the Spanish (*r* = − 0.35to-0.75) [61], German (*r* = − 0.48to-0.78) [43] and Chinese (*r* = − 0.25to-0.75) [44] version of ODI. On the contrary, the Brazilian ODI version has shown positive correlation (*r* = 0.19–0.83) with all domains of SF-36 [20]. These findings suggest that ODI-U shows positive moderate correlation with VAS disability and VAS pain while a moderate negative correlation between ODI-U and SF-36 domain.

## Limitations

- The first limitation was responsiveness; to detect change over time was not measured as no treatment was given to the patients.
- In order to evaluate test-re-test reliability, a short interval of 3 days was used to ensure patient’s condition remain same. Therefore, memory effects in this study could not be ruled out completely.
- Data was collected from outpatient physiotherapy clinics only. The reliability and validity of ODI-U was not tested in other populations such as lumbar canal stenosis, Lumbar fusion, surgical stabilization, decompression surgeries, etc. Therefore the results may not be generalized to patients suffering from back pain as well as to inpatients.

## Strengths

- The primary strength of study was that, by using pre-defined hypotheses the psychometric properties of the ODI-U were analyzed.
- Up to the authors’ knowledge, it was the only research study who has measured the item-total correlation for confirming the internal consistency of the scale.

## Conclusion

It is concluded that ODI-U is psychometrically reliable and valid questionnaire to assess level of disability in patients with lumbar radiculopathy. It has simple and easy language that can be understood easily by the Urdu-speaking patients. Therefore, the clinicians and researchers may use ODI-U to evaluate the back disability in Urdu-speaking patients having lumbar radiculopathy.

## Supplementary Information

**Additional file 1: Appendix I.** Oswestry Disability Index (ODI) version 2.1a.

## Data Availability

All data generated or analysed during this study are included in this published article [and its supplementary information files].

## Abbreviations

LBP: Low back pain
COSMIN: COnsensus-based Standards for the selection of health status Measurement Instruments
ICC: Intra-class correlation coefficient
RMQD: Roland Morris Disability scale
ODI: Oswestry disability index
ODI-U: Urdu version of the Oswestry disability index
SD: Standard deviation
KMO: Kaiser-Meyer-Olkin
SDC: Smallest detectable change
SEM: Standard error of measurement
VASdisability: Visual analogue scale for disability
VASpain: Visual analogue scale for pain
