# Supplementary material for: Urdu version of Oswestry disability index; a reliability and validity study
Source: BMC Musculoskelet Disord. 2021 Mar 29;22:311. doi: 10.1186/s12891-021-04173-0 (PMC8008691; doi:10.1186/s12891-021-04173-0)
Supplement: Supplementary file 1 — Additional file 1: Appendix I. Oswestry Disability Index (ODI) version 2.1a. [file 12891_2021_4173_MOESM1_ESM.docx]

**URDU VERSION OF OSWESTRY DISABILITY INDEX; A RELIABILITY AND VALIDITY STUDY**

**Corresponding Author:**

Fareeha Amjad

Email: [fari_fairy22@yahoo.com](mailto:fari_fairy22@yahoo.com), [fareeha.amjad@uipt.uol.edu.pk](mailto:fareeha.amjad@uipt.uol.edu.pk)

Phone number: 0334-3372779

University Institute of Physical Therapy, Faculty of Allied Health Sciences, The University of Lahore, Lahore, Pakistan.

**Co-Authors:**

- **Mohammad A. Mohseni-Bandpei**

Email: [mohseni_bandpei@yahoo.com](mailto:mohseni_bandpei@yahoo.com)

University Institute of Physical Therapy, Faculty of Allied Health Sciences, The University of Lahore, Lahore, Pakistan.

Pediatric Neurorehabilitation Research Center, University of Social Welfare and Rehabilitation Sciences, Tehran, Iran.

- **Syed Amir Gilani**

Email: [profgilani@gmail.com](mailto:profgilani@gmail.com)

Dean Faculty of Allied Health Sciences, Director; Directorate of International Linkages, University of Lahore, Lahore, Pakistan

- **Ashfaq Ahmad**

Email: [ashfaaqpt@gmail.com](mailto:ashfaaqpt@gmail.com)

Associate Dean Faculty of Allied Health Sciences, Head of Department University Institute of Physical Therapy, The University of Lahore, Lahore, Pakistan.

- **Muhammad Waqas**

Email: [drwaqasfayyaz@gmail.com](mailto:drwaqasfayyaz@gmail.com)

Faculty of Allied Health Sciences, Head of Department University Institute of Physical Therapy, The University of Lahore, Lahore, Pakistan.

- **Asif Hanif**

Email: asif.hanif@ahs.uol.edu.pk

University Institute of Physical Therapy, Faculty of Allied Health Sciences, The University of Lahore, Lahore, Pakistan.

**Appendix I**

**Oswestry Disability Index (ODI) version 2.1a**

This questionnaire is designed to give us information as to how your back (or leg) trouble affects your ability to manage in everyday life.

Please answer every section. Mark one box only in each section that most closely describes you today.

Section 1 - Pain intensity

o (0) I have no pain at the moment.

o (1) The pain is very mild at the moment.

o (2) The pain is moderate at the moment.

o (3) The pain is fairly severe at the moment.

o (4) The pain is very severe at the moment.

o (5) The pain is the worst imaginable at the moment.

Section 2 - Personal care (washing, dressing, etc.)

o (0) I can look after myself normally without causing extra pain.

o (1) I can look after myself normally but it is very painful.

o (2) It is painful to look after myself and I am slow and careful.

o (3) I need some help but manage most of my personal care.

o (4) I need help every day in most aspects of self-care.

o (5) I do not get dressed, wash with difficulty and stay in bed.

Section 3 - Lifting

o (0) I can lift heavy weights without extra pain.

o (1) I can lift heavy weights but it gives extra pain.

o (2) Pain prevents me from lifting heavy weights off the floor but I can manage if they are  conveniently positioned, e.g. on a table.

o (3) Pain prevents me from lifting heavy weights but I can manage light to medium weights if  they are conveniently positioned.

o (4) I can lift only very light weights.

o (5) I cannot lift or carry anything at all.

Section 4 - Walking

o (0) Pain does not prevent me walking any distance.

o (1) Pain prevents me walking more than one mile.

o (2) Pain prevents me walking more than a quarter of a mile.

o (3) Pain prevents me walking more than 100 yards.

o (4) I can only walk using a stick or crutches.

o (5) I am in bed most of the time and have to crawl to the toilet.

Section 5 - Sitting

o (0) I can sit in any chair as long as I like.

o (1) I can sit in my favorite chair as long as I like.

o (2) Pain prevents me from sitting for more than 1 hour.

o (3) Pain prevents me from sitting for more than half an hour.

o (4) Pain prevents me from sitting for more than 10 minutes.

o (5) Pain prevents me from sitting at all.

Section 6 - Standing

o (0) I can stand as long as I want without extra pain.

o (1) I can stand as long as I want but it gives me extra pain.

o (2) Pain prevents me from standing for more than 1 hour.

o (3) Pain prevents me from standing for more than half an hour.

o (4) Pain prevents me from standing for more than 10 minutes.

o (5) Pain prevents me from standing at all.

Section 7 - Sleeping

o (0) My sleep is never disturbed by pain.

o (1) My sleep is occasionally disturbed by pain.

o (2) Because of pain I have less than 6 hours sleep.

o (3) Because of pain I have less than 4 hours sleep.

o (4) Because of pain I have less than 2 hours sleep.

o (5) Pain prevents me from sleeping at all.

Section 8 - Sex life (if applicable)

o (0) My sex life is normal and causes no extra pain.

o (1) My sex life is normal but causes some extra pain.

o (2) My sex life is nearly normal but is very painful.

o (3) My sex life is severely restricted by pain.

o (4) My sex life is nearly absent because of pain.

o (5) Pain prevents any sex life at all.

Section 9 - Social life

o (0) My social life is normal and causes me no extra pain.

o (1) My social life is normal but increases the degree of pain.

o (2) Pain has no significant effect on my social life apart from limiting my more energetic  interests, e.g. sport, etc.

o (3) Pain has restricted my social life and I do not go out as often.

o (4) Pain has restricted social life to my home.

o (5) I have no social life because of pain.

Section 10 - Travelling

o (0) I can travel anywhere without pain.

o (1) I can travel anywhere but it gives extra pain.

o (2) Pain is bad but I manage journeys over two hours.

o (3) Pain restricts me to journeys of less than one hour.

o (4) Pain restricts me to short necessary journeys under 30 minutes.

o (5) Pain prevents me from travelling except to receive treatment

Total Score=

Your ODI = %

ODI % = Total score/5 x Number of questions answered x 100

ODI © Jeremy Fairbank, 1980. All Rights Reserved.
